# Enjoymeter for Exploring Micro-Scale Changes in Foreign Language Enjoyment

**DOI:** 10.3389/fpsyg.2022.882748

**Published:** 2022-05-16

**Authors:** Rouhua Wang

**Affiliations:** School of Foreign Languages, Changchun Institute of Technology, Changchun, China

**Keywords:** foreign language enjoyment, enjoymeter, micro-scale, dynamics, English as a foreign language

## Abstract

With the popularity of positive psychology research in second language acquisition since 2012, foreign language enjoyment (FLE) has attracted many researchers in this domain. Several innovative quantitative and qualitative research methods have been used so far to explore FLE. However, given the dynamic nature of FLE, the development of innovative tools can contribute to the exploration of the micro-scale dynamics of FLE. This study aims to introduce *enjoymeter* as one of these innovative tools and provide an example for its use in a foreign language learning setting. To do this, the application of enjoymeters in the exploration of the dynamics of FLE in an English as a foreign language course in China is explained. The enjoymeter data along with interviews were collected in three sessions of the course and were analyzed *via* thematic analysis. The findings indicated that moments of enjoyment emerged in terms of both private FLE and social FLE in these three sessions. Also, the use of enjoymeter indicated that it can enable researchers to map the dynamics of FLE session by session and even within each session of the course. The pedagogical implications of the use of enjoymeters in foreign language classes are discussed and future directions are explained.

## Introduction

The recent popularity of positive psychology (PP) in second language acquisition (MacIntyre and Mercer, 2014; MacIntyre et al., 2016), in tandem with the growing interest in the complex dynamic system theory (CDST), has been accompanied by a new line of research on the dynamics of PP variables, such as foreign language enjoyment (FLE). FLE has been proved to be an ever-changing construct that emerges under the influence of different internal and external contextual factors in the ever-changing complex world of language learners (refer to Dewaele and Dewaele, 2017, 2020; Elahi Shirvan et al., 2020, 2021). Thus, researchers in the field of second language acquisition (SLA) have been eager to examine the dynamics of this variable under the influence of such factors as well. The inherent dynamicity of FLE is further evident in its two components at the core of the definition, the social and private components. The former is influenced by friendly and supportive peers and teachers (Khajavy et al., 2018) and the latter by the private dimension focused on personal progress and self-development (Dewaele and MacIntyre, 2014, 2016; Pavelescu and Petric, 2018). With this dynamic line of inquiry in mind, Shao et al. (2019) summoned up researchers to explore emotions that affect EFL learning by contriving original research design, approaches, and tools as well as adapted ones from similar disciplines. Moreover, Shao et al. (2019) invited researchers in the field of SLA to integrate research on achievement emotions with research on emotions in this field. Thus, inspired by these invitations, and in line with the dynamic perspective toward FLE, the present research aimed to introduce a CDST compatible tool, *enjoymeter*, using it for the explorations of the dynamics of FLE over time.

## Literature Review

### Foreign Language Enjoyment

Positive psychology found its way into SLA research in 2012 by MacIntyre and Gregersen. The new line of research on these emotions represents a drastic change from a language anxiety-based line of inquiry in the second language acquisition domain to the acknowledgment research on positive emotions, influenced by the broaden-and-build theory of positive emotions (Fredrickson, 2001). This theory posits that an individual's momentary thought-action repertoire can be broadened by positive emotions (Fredrickson, 2001). Since then, several studies have explored positive emotions in acquiring L2 with a focus on foreign language enjoyment (e.g., Dewaele and Dewaele, 2017; Kruk et al., 2022; Li and Wei, 2022). The simplest way to define FLE is by comparing and contrasting it against pleasure (Dewaele and MacIntyre, 2014). Enjoyment is viewed as the pleasant sense that one develops from trespassing homeostatic limits and reaching for new experiences, particularly in the face of challenging tasks. In contrast, pleasure refers to only a desirable feeling one has when the homeostatic needs (including physical comfort, sex, or hunger) are satisfied adequately (Dewaele and MacIntyre, 2016). In fact, enjoyment is defined as “a sense of novelty and accomplishment” (Seligman and Csikszentmihalyi, 2000, p. 46) that contributes to permanent health and personal growth (Seligman and Csikszentmihalyi, 2000). From the perspective of control-value theory of achievement emotions, enjoyment can be defined as a positive activating emotion that is tied to students' perceptions of high control over, and positive value of, current achievement-related activities (Shao et al., 2020).

There has been ever-increasing interest, in FLE research, in the dynamic nature of the construct and its variation intra-individually or within groups (e.g., Dewaele and Dewaele, 2017; Boudreau et al., 2018; Elahi Shirvan and Taherian, 2021). More specifically, such investigations have been inspired by CDST (Ushioda, 2015; Larsen-Freeman, 2016), which has influenced SLA research on individual difference (ID) variables for at least a decade. There is increasingly more evidence for the ever-changing learner and teacher emotions and the inherent dynamicity influenced by several internal and external factors hand-in-hand that can account for long-term variations (Jiang and Dewaele, 2019; Elahi Shirvan and Talebzadeh, 2020). The current emphasis on IDs in SLA is intensified by viewing language learners more than ergodic ensembles. It implies that the average level of an ID does not necessarily represent the individual level of that ID (Lowie and Verspoor, 2019). The new line of research on FLE is influenced and enriched by the innovative research methodologies in CDST (for more information refer to Hiver and Al-Hoorie, 2019). In other words, using these methodologies, a body of research was begun to approach the dynamic dimensions of FLE (e.g., Dewaele and Dewaele, 2017, 2020; Boudreau et al., 2018; Elahi Shirvan and Talebzadeh, 2018a,b, 2020; De Ruiter et al., 2019; Talebzadeh et al., 2019; Elahi Shirvan and Taherian, 2021).

### Complex Dynamic System Theory in SLA

The prominence of CDST in SLA research has been influenced by social disciplines which provide evidence that most of the important issues of our time are complex and dynamic and must be viewed from this perspective (Capra and Luisi, 2014). Larsen-Freeman (1997) was the first to propose that SLA issues should be dealt with explicitly from a complex dynamic perspective. Since then, many researchers have held CDST in SLA to assess different domains of the field, such as language development or acquisition (de Bot, 2008; Verspoor et al., 2008; Ellis and Larsen-Freeman, 2009), language ecology (Kramsch and Whiteside, 2008), language attrition (Schmid, 2013), language change (Kretzschmar and Kretzschmar, 2015), language development (Ke and Holland, 2006; Mufwene, 2017), and psychology of language learning and teaching (Gkonou et al., 2016).

When CDST entered SLA, the focus in the latter field was shifted to continuous system change and interconnectedness. CDST has provided researchers with a new perspective that challenges the primary conceptions of scientific inquiry. For instance, in SLA, it has questioned conventional thoughts concerning language and its development (Hiver and Al-Hoorie, 2019; Hiver et al., 2021), such as the composition of language as a static system of rules. From the CDST perspective, language learning is dynamic and emerges or self-organizes into different patterns out of the interaction between different context-bound factors. CDST adopts an ecological view, mainly rooted in poststructuralism, in which both temporal and spatial context as well as intra- and inter-individual differences play a pivotal role in the development of language-related constructs (Larsen-Freeman, 2020). That is, learners are not separated from their learning process and the situated ecology of their learning. This is due to the fact that complex systems are self-organizing, co-adaptive, and interconnected and therefore often viewed as trans-disciplinary (Hiver and Al-Hoorie, 2019; Hiver et al., 2021).

In the CDST framework, variability is a pivotal property that differentiates learners in the language development process. More specifically, the identified differences among learners are seen as inter-individual variability (Verspoor et al., 2008) based on which they follow their own path in a self-organizing system (De Bot et al., 2005). Different patterns of variability reflect different kinds of learning trajectories and developmental patterns (Verspoor et al., 2008). It should be noted that each learner also experiences variability in his or her own developmental process, referred to as intra-individual variability, which indicates each individual learner's uniqueness in learning different aspects of a second or foreign language.

### FLE From a CDST Perspective

A body of research has attested to the complexity, dynamicity, and multifacetedness of FLE (Dewaele and Dewaele, 2017; Elahi Shirvan and Taherian, 2020, 2021). From the perspective of CDST, it seems that previous works of research assessed FLE from a person-oriented perspective, also known as the idiographic point of view (Jiang and Dewaele, 2019; Elahi Shirvan and Taherian, 2020; Elahi Shirvan and Talebzadeh, 2020; Elahi Shirvan et al., 2020) or from a variable-oriented perspective, also known as nomothetic approach (Dewaele and Dewaele, 2017; De Ruiter et al., 2019). Yet, some employed a mixed method (Dewaele and MacIntyre, 2019). It should be noted that the nomothetic perspective emphasizes the overall mean trajectory of all cases (Hiver and Al-Hoorie, 2019). On the other hand, the idiographic perspective is focused on the unique and idiosyncratic trajectories of each individual (Hiver and Al-Hoorie, 2019) as reviewed below. The pioneering attempt to trace the dynamic development of FLE based on a nomothetic approach was made by Dewaele and Dewaele (2017) in some pseudo-longitudinal research to explore FLE variation over time, with a dynamic approach. A total of 3 classes of students were distinguished for age and showed a slight increase in FLE over time. Further regression analyses revealed that few teacher-centered and learner-internal factors predicted FLE at the outset and at the end of high school compared to junior high school. These results implied that the sources of FLE are dynamic and vary between 12 and 18 years of age. In the same line of research, Dewaele and MacIntyre (2019) employed a mixed method to explore the influence of learner-external and learner-internal variables on FLE and anxiety. Confirming evidence was found for the negative correlation between FLE and anxiety. Furthermore, multiple regression analyses indicated that teacher-centered factors, including attitudes to the teacher, teacher's friendly behavior, and joking highly predicted FLE.

With a focus on the dynamic quality of emotions, De Ruiter et al. (2019) explored the within-individual fluctuations of FLE situated in the changing ecology of class learning. They observed the emergence of recurring patterns of students' anxiety and enjoyment as well as teacher support during these interactions that draw attention to the self-patterning quality of such teacher–student relationships. Besides, based on the peculiar nature of the emergent patterns of FLE, they suggested that the conventionally perceived positive link betwixt students' emotions and teacher's supportive role may be extended to real-life and real-time procedures. Besides, Dewaele and Dewaele (2020) investigated to what extent FLE changes at a single moment when learners face two different teachers for the same L2. They found that the participants experienced significantly more FLE in the presence of their main teacher.

It was strongly associated with a more positive perception of the main teacher and his/her more use of foreign language in class. As the existing literature shows, cross-sectional studies are dominant in the field and there is a significant lack of longitudinal exploration of FLE to provide insights into the dynamic nature of this emotion Dewaele et al. (2019). With this gap in mind, Elahi Shirvan and Taherian (2021) *via* a longitudinal study traced the dynamicity of undergraduates' FLE over an entire course of general English language. Given the growing attention to the dynamic aspects of FLE, the need for the introduction and application of CDST-compatible tools is felt more than before (refer to Hiver and Al-Hoorie, 2019). In this study, the procedures for the application of enjoymeters for the expansion of the dynamic line of inquiry on FLE with an example study are explained in detail. It should be mentioned that enjoymeters were used specifically to answer the following research question:

How, and under the influence of what factors, does enjoyment change in three sessions of an English as a foreign language course?

### Participants and Procedures

The case study was done in an intermediate-level EFL course (B2) in a Chinese private language institute. Since the data collection procedure was predictably time-consuming (it took 2 months), it was essential to include a participant who could wholeheartedly volunteer to take part in the research. The aim was to ensure the accuracy of the data collected. A written statement of the purpose of the research and data collection phase was provided for the participant in the class. A female student showed interest in participating in the study. She signed a letter of informed consent to take part. She was assured that she could withdraw from the study anytime she wanted. When one session (of the class) ended, she was met in person and was provided with more details on the research project. She shared the Chinese culture and academic grounding and was an intermediate proficiency level learner of the English language as substantiated by the Oxford Placement Test. She is named henceforth as Mary for ethical considerations. At the time of the study, Mary was 19 years old. With respect to the focused language course in this study, I should mention that it was a course of an intermediate level of English as a foreign language in a private language institute. The students in the class were involved in doing different activities for the improvement in their reading, writing, listening, and speaking skills in the English language while receiving the required instruction for doing them from their course teacher. Given the nature of each task, they were supposed to do some of the classroom tasks individually and some others *via* pair work and group work.

### Enjoymeter in Data Collection

The enjoymeter was designed in the light of longitudinal classroom-scale investigations done by Gardner et al. (2004) and Waninge et al. (2014), who used an anxometer and motometer, respectively (refer to Appendix). At first, an A4 piece of paper was submitted to each subject with 20 enjoymeters. They looked like thermometers in appearance and ranged from 0 (minimum enjoyment) to 10 (maximum enjoyment). The participant was instructed to mark her level of enjoyment *via* the enjoymeters by drawing a horizontal line on the enjoymeter after every 10 min. In-class enjoymeters were collected time-to-time during which the participant was expected to mark the level of enjoyment she felt while using English in class.

The intervals were 10 min long. Each classroom session was also audio-recorded to trace the fluctuations of the participant's perceived enjoyment at regular intervals. The participant was supposed to mark her enjoyment ratings in the enjoymeters for every 10 min and comment on the level of enjoyment she experienced along with the underlying factors affecting those levels. The whole process occurred during three separate lessons 5 weeks apart from each other. A decision was made to begin the first phase of the enjoymeter data collection in the 3rd week of the course as the class was more accustomed to the instructional setting. Also, a number of open-ended interviews were held in the participant's first language at the end of each session of the course. The purpose of these interviews was to gain further information with regard to the marked levels of enjoyment in each session enjoymeters and the relevant written comments for them regarding the underlying factors of momentary changes in enjoyment.

### Data Analysis

A qualitative analysis was followed to code the transcribed data of interviews along with the comments written in each session enjoymeters line by line not to miss any aspect of the collected data and account for any abrupt point raised during the analysis. After the coding procedure, the extracted codes were integrated or further developed until well-organized categories were made and the data saturation occurred. For theme extraction, the codes and categories were analyzed with an emphasis on the momentary variations of the participant's enjoyment patterns *in situ*. The codes related to *enjoyment of teacher support, enjoyment of peer support*, and *enjoyment of doing language learning tasks* were categorized as *the dynamics of social FLE*. Also, the ones related to self-derived experiences of enjoyment were categorized as *the dynamics of private FLE*. Furthermore, the coding process was checked by an expert in the field, which led to a high level of inter-coder agreement. For the purpose of this study, the micro-changes of Mary's enjoyment in only three sessions of the course (one at the beginning of the course, one in the middle, and one near the end of the course) are explained.

## Results

### Mary's Moments of High Enjoyment

**Figure 1:**
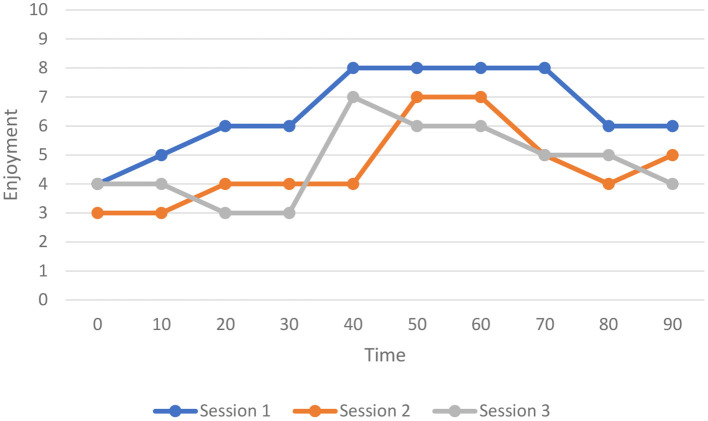
Dynamics of Mary's enjoyment in EFL learning.

Mary's enjoymeters revealed signs of high enjoyment within her class activities, the nature of which needs to be discussed in detail to reveal the possible contributing factors to her enjoyment. The range of her enjoyment varied from 3 to 8 (in three sessions) in every 10-min duration of class-related events. According to Figure 1, no single rating reached the maximum enjoyment of 10. Below is the trajectory of Mary's dynamicity of enjoyment in three times of data collection (three sessions).

It is noteworthy that the first session of data collection was actually in the 3rd week of the course. The second session (of data collection) was in the 5th week of the course and the third session was in the 7th week of the course. In other words, session one belonged to the opening of the course, session two to the middle of course, and session three to the end of the course. Considering the 2-week time interval between the sessions, as expected, three different lessons were taught in these sessions.

### Enjoyment in Session #1

In the 1st session (the 3rd week of the course), Mary began the class with an enjoyment level of slightly below the average. Within the first 20 min of class, her enjoyment was increasing. Then, it remained constant for 10 min until it rose again to its maximum state (grade 8). For 1/2 h (between the 40th and 70th min of class time), her enjoyment remained high. Then, it was reduced until the class ended. A short look at the trajectory of changes shows that the class time can be viewed in three parts in terms of the level of changes in Mary's enjoyment. The trend is rising in the first part of the class time, then remained constant for the second part of the class time (middle of class), and then was falling until the end of the class time. This orientation of enjoyment to rise and fall in a single session is of interest from a dynamic viewpoint. Variations in the degree of enjoyment inside every session can reveal interesting points about the environment of class, quality of tasks/activities involved, and student–student and teacher–student interactions. Up to this stage, the data analysis showed that Mary enjoyed the whole session from beginning to the end yet to different degrees.

#### The Dynamics of Mary's Enjoyment in Terms of Private FLE

The data retrieved from Mary's enjoymeter showed that the moments of her enjoyment in the first session emerged as both private FLE and social FLE. This means that the fluctuations in both the private FLE including those about the “self,” and the social FLE including those concerning “self–teacher,” “self–peers,” along with “self–peers–teacher” marked Mary's enjoymeter commenced from session one. First, many parts of her moments of high enjoyment dealt with her private FLE, including her positive attitude toward language learning within the class events, especially at the outset of the course. She had not begun the course with a neutral or negative attitude to language learning. Her positive attitude to language learning could have contributed to her about-the-average level of enjoyment at the beginning of session one. Below is a relevant account from her interview:

*Some of my classmates drag themselves to class. They keep nagging every session sometimes even from the beginning to the end. I see no point in nagging about what I have chosen to go through. I feel the desire within to learn English and try my best not to miss even one session*.

The above-mentioned account shows that Mary was internally motivated to learn the language and it can be at least part of the reason why she enjoyed the class. Private FLE was also highlighted in Mary's enjoymeters during personal development, or conception of satisfactory achievement in an online classroom (in session one). This is supported by her following experience:

*In this session, when the teacher asked the students to read the vocabulary in passages (almost in the middle of the session), I also actively cooperated. I shared the audio-recorded loud-reading of the vocabulary in our chat group and was admired by our teacher. Before the online classes, I was too embarrassed to read English in the presence of everyone in class. Now I could happily do that, and I felt confident in English. I thought I had to work harder to get better in English*.

It is evident from Mary's own admitted satisfaction that her perceived personal development must have greatly contributed to her enjoyment in the middle part of session one. She continued:

*After the more cooperative tasks, near the end of the class, my classmates insisted that the teacher did not teach anything new and let them just answer the speaking questions on the next page and skip the writing task. I preferred to continue the cooperative task but the teacher went as she wished*.

It seems that changing the class activity to a less cooperative one and getting down to a simpler and less motivating task reduced Mary's level of enjoyment by the end of the class time.

#### The Dynamics of Mary's Enjoyment in Terms of Social FLE

The social FLE aspect of enjoyment was also manifested in Mary's experience of language learning in session one. Regarding factors related to “self–peers” accounting for her momentary perceived enjoyment, she mentioned the chances of communicating with the same students during the cooperative vocabulary task that was run in class in the middle part of this session. She found the chances of expressing herself and exchanging ideas with peers very enjoyable and the least stressful. See below:

*I enjoyed sharing my notes with my classmates; they are at the same level as I am; I felt no fear of admitting to my mistakes in pair-work. They make many mistakes too and we freely comment on each other's notes*. She mentioned a high sense of joy when she could establish closer social bonds with her peers during group discussions as well as group work in the online setting. She explained: “*My classmates appreciated me for taking notes in class; that made me happy and I really enjoyed it”*.

It is evident that her teacher played a key role too in the sense of enjoyment she felt. A number of “self–teacher”-related variables that could affect the joyful moments Mary experienced in session one can be pinpointed as the teacher's approval/encouragement and individual attention/recognition, particularly oral approval by her teacher on the Internet. Mary enjoyed the teacher's encouragement:

*Before the cooperative task, the teacher checked my class notes and praised my job. That gave me a feeling of both pleasure and achievement. It rose my good feeling suddenly. It was so encouraging and motivating for me to do the class activity even better next time and grow interest in learning English language. After that I was very eager and confident to share my notes with friends*.

Evidently, her teacher's encouraging feedback on Mary's notes managed to increase her level of enjoyment from the very beginning of the session to the middle when the student–student cooperative task began and Mary's enjoyment reached its highest level. The teacher helped her to feel well-recognized in class, especially in the presence of peers.

Overall, it can be concluded that the increasing, then constant (at the maximal level), and then decreasing trends of Mary's enjoyment during session one contained signs of both private and social aspects of FLE. More specifically, the changing intensity of enjoyment was a function of internal factors (e.g., internal motivation to learn the language) and social factors (e.g., cooperation with peers or the teacher).

### Enjoyment in Session #2

In the 2nd session of data collection (the 5th week of the course), Mary began the class at a lower-than-average level of enjoyment. For the first 10 min of class, her enjoyment did not vary. Only then, it rose slightly for 10 min and remained steady for the next 20 min of class time. When almost half of the class time was gone, her enjoyment rose suddenly by 3 degrees. This sudden increase was followed by a steady status until 60th min of the session. Next, there was a sharp decrease in enjoyment for 3 degrees for 20 min before it rose slightly (for 1 degree) until the class ended. As Figure 1 shows, a quick look at the trajectory of changes in enjoyment shows a rising trend until the middle of class time and then a falling trend until the end.

#### The Dynamics of Mary's Enjoyment in Terms of Private FLE

Mary's interview content was reviewed to see what factors contributed to this rise and fall of the emotion. A review of the class activities in the second session showed that the beginning of the session was concomitant to a review of the previous session's assignments which were more teacher-directed and less peer interaction was involved. It should be mentioned that checking the tasks one by one created chances of correcting mistakes and learning the right answers, which managed to slightly and gradually increase Mary's enjoyment. She slightly felt enjoying her own recognition of weaknesses, which can be seen as a private FLE aspect. Below is part of her own description:

*There were lots of exercises to do at the beginning of class. They took almost half an hour. We did not work in pairs or groups. The teacher called us one by one to read our answers. I could see my mistakes and could correct them. I had the chance for correcting my answers without others knowing about them. My face was saved. The teacher gave all of us almost an equal chance to check the answers*.

From Mary's account, it can be inferred that her gradually rising level of enjoyment was due to her perceived personal development, self-correction, and face-saving performance. These contribute to the private FLE for sure, as they are defined around the self. Self-teacher, self-peer, and peer–peer interactions seem to have been kept to a minimum (probably deliberately to save time).

#### The Dynamics of Mary's Enjoyment in Terms of Social FLE

After the review section of the class was over, there seems to be a sharp increase in Mary's enjoyment within the next 10 min of class. Mary's interview revealed that this sudden rising trend of enjoyment emerged when the teacher introduced the warm-up section of the new lesson by asking students open-ended questions to tap into their old memories and life experiences (the topic of the lesson being the “Travel”). The teacher's warm-up managed to engage all students, as Mary admitted:

*A nice topic followed and we were all eager to say something about it. I raised my hand to talk about my last vacation. There was still no pre-teaching of vocabulary, and we could use our own words, so we could talk freely*.

It appears that Mary's teacher could manage to motivate the students by initiating teacher–student and student–student talks. Moreover, the students could volunteer to share their experiences with the class. Voluntary participation in class activities could have enhanced Mary's enjoyment and her willingness for more free discussions. Furthermore, it seems that the sources of the high-level enjoyment Mary experienced in this part of the class time were related to the self-peer–teacher interactions which appeal more to the social FLE aspect. Here again, evidently, the teacher played a key role in increasing her level of enjoyment. Without an appropriate, cooperative, and thought-provoking warm-up, the teacher could have only jumped to the instruction (of new content) phase, missing students' whole-person involvement in the learning process. But she managed to push the students' boredom away and, instead, attract their attention to the topic of the new lesson and let them enjoy their sharing of thoughts and experiences. Students' role during this warm-up activity seems to have been less creative yet more active and cooperative, pointing to Mary's overall high level of enjoyment (evidenced by their voluntary participation in the task).

Mary kept her enjoyment high throughout the next 10 min when new related vocabulary was also taught using a mind map technique by the teacher. Mary mentioned that she still enjoyed it as much when she began to learn new words that she desperately needed to know to express ideas (about the travel topic) she already had in mind. The falling trend of Mary's enjoyment followed from the silent reading task for the passage within the textbook. Mary explained that for the next 20 min (during which the reading passage was dealt with), there was no self-peer, self-teacher, peer–peer, or peer–teacher interactions. In other words, Mary's enjoyment seems to have decreased for 20 min due to the lack of any contribution to her private FLE and social FLE aspects. She also pointed out that she preferred her teacher to read the text line-by-line, so that she could follow and comment on the structure or content of the lesson. She felt a need to have someone hear her voice while reading the passage aloud and correcting her mispronunciation too. Yet, there was no such activity involved. Mary was feeling more and more losing control of the passage content and its meaning. Here, it seems that she felt she did not have the teacher's support anymore, neither that of her peers. Thus, the level of her enjoyment was continuously falling, until the last 10 min of class when the students were assigned to discuss the answers to the reading comprehension questions together in pairs and then report it to class. Though the time was limited, the teacher sought to run an activation phase of the teaching to make students active. It seems that this last part of the lesson managed to slightly increase Mary's enjoyment, as she described below:

*The reading task is not cooperative. It took us much time to read and get the gist of the text. We were tired too. We were encouraged to use a dictionary too, which was very time-consuming. But the group-work comprehension check part was good. I liked it. It changed our mood before the class ended*.

Here, it seems that Mary experienced a decreasing trend of enjoyment in the latter part of the class due to the nature of task involved and the less cooperation and interaction it required. What decreased her enjoyment level was the lack of self-peer and self-teacher interactions and the fading social aspect of FLE. More specifically, what fed into her slightly rising enjoyment in the last 10 min of class time was the resort to the cooperative and thought-sharing part of the task which appealed to her social FLE. It is evident that to Mary the self-peer and self-teacher interactions mattered more than self-textbook interaction, the former appealing to the private and the latter to the social aspect of her enjoyment.

### Enjoyment in Session #3

In the 3rd session (the 7th week of the course), Mary began the class with an enjoyment level the same as session one. The intensity of her enjoyment was slightly below the average (<5). For the first 10 min, her enjoyment did not change. Afterward, it began to decrease until it remained stable at a lower degree than the outset of the class for 10 more min. There was a sudden increase (for 4 degrees) in her enjoyment after the first 1/2 h of class. Yet, after 10 min, Mary's enjoyment began to decrease gradually until the end of the session. Overall, her level of enjoyment in session three was lower than that of session one (as the line graph shows); yet, the fluctuations were more. The range of enjoyment she felt during this session was between 3 and 7.

#### The Dynamics of Mary's Enjoyment in Terms of Private FLE

The session opened with a review of the questions formerly included in the quiz of the previous session. Mary thought that the task would go on with the recitation of the writing assignments she had prepared at home. She hoped to find a chance to voluntarily read out her writing task response and receive feedback from the teacher. However, things did not go as she expected, see below:

*I was losing my initial interest as I felt the chances for reading out my writing homework were gone and they were replaced by checking the quiz content. The task was not enjoyable to me as I really preferred to have my writing read aloud instead and assess myself*.

Here, it appears that Mary's lowered enjoyment compared to the outset of the session was caused by her unmet expectations. As her account shows, she expected to enjoy self-assessment through a voluntary read-aloud task which was replaced by a quiz-check task. In fact, she felt deprived of the contribution to her private FLE, around the self-development. Though she flowed the class procedure, things did not appeal to her.

#### The Dynamics of Mary's Enjoyment in Terms of Social FLE

The whole mood changed after 1/2 h of the class time when the new lesson began and the students were asked to brainstorm together about the conversation topic “friendship.” While students were sharing their opinions, the teacher was actively using the board to give useful hints to students and guide their conversations. Mary found the conversation topic intriguing, and also, she appreciated her teacher's role in not standing aside and, instead, providing continuous feedback to students during the cooperative task. See a relevant account below:

*Friendship was a topic we all liked to talk about. We all had something to share, and were eager to talk. The teacher was predicting the words we needed and wrote them on the board so that everyone could see*.

Another factor feeding into her instances of high enjoyment in the mid-part of the third session of data collection (the 7th week) was her prevalent use of English with her classmates (peers). Mary perceived these moments as enjoyable as she enjoyed the chances of classroom communication in online chat rooms with her peers. Below is her own comment:

*Overall, I enjoyed the conversation exchange; there was a friendly mood around. All my friends were rather open up. We could chat in our Telegram group in English. My friends' self-made videos were very amusing. We had a friendly group discussion*.

The 10-min highly enjoyable peer–peer talk was followed by individual reports to class, which seems to have gradually decreased Mary's enjoyment. Susceptible to her classmates' judgment of her speech in this part of the class (the second half of class time) (the 7th week), Mary went through the moments of decreasing enjoyment. The lowering trend of enjoyment continued until the end of the session when the individual report task was followed by the task of answering the comprehension questions after the conversation. They were supposed to be written down, which Mary found rather boring. It is evident that the anxiety-provoking individual report task contributed to the decreasing trend of Mary's enjoyment. She found the task face-threatening and did not perceive it appealing. In other words, the self-teacher interaction in this task neither contributed to her private nor her social FLE.

Overall, it can be observed that Mary's enjoyment in session 3 was lower than her enjoyment in session 1 during the whole class time. It also had more fluctuations. Compared to session 2, in some parts of the class time, the expressed enjoyment was higher, and in some other parts, it was lower. There were intersections too. Yet, the range of variations of her enjoyment was the same in the two sessions (#2 and 3). As Mary's interview showed, the out-of-class experiences, such as her home conditions, were a major factor accounting for the low degrees of her enjoyment moments in the primary minutes of the second half of the third session of the data collection (7th week). About this, Mary described:

*I had a row with my mom…at the beginning of the class, I was carried away by the mood of class and almost forgot all about it, but later on in the end of the class when I was tired, all the negative thoughts came back to me. Maybe that was because the class was being ended and I had to join the family gathering again*.

It shows that though such personal experiences in life were beyond the immediate context of the class, they managed to influence (adversely affect) Mary's enjoyable moments in class.

## Discussion

In line with the purpose of study, as an innovative measurement instrument, the enjoymeter was used to trace the dynamicity of enjoyment that Mary, an English language learner in China, experienced throughout an EFL course. A total of three specific sessions for data collection were selected, one at the beginning of the course, one in the middle, and one within the final weeks of the course. The trajectory of changes in her FLE showed significant fluctuations within and between the sessions. This finding is in line with a number of studies attesting to the dynamicity of FLE and its variations in and across instructional sessions (e.g., Dewaele and Dewaele, 2017; Boudreau et al., 2018; Shao et al., 2020; Elahi Shirvan and Taherian, 2021). The research findings proved that the new instrument, the enjoymeter, was capable of capturing the dynamicity of FLE and being effectively used in longitudinal studies of the construct. This feature makes this study complementary to the line of longitudinal studies of FLE which formerly used conventional qualitative and quantitative methods of data collection and analysis (e.g., Dewaele and Dewaele, 2017; Boudreau et al., 2018; Elahi Shirvan and Talebzadeh, 2018a,b; Talebzadeh et al., 2019; Shao et al., 2020).

The enjoymeter data showed that, overall, Mary experienced a higher level of enjoyment during the first phase of data collection (i.e., at the beginning of the course). Her enjoyment was lower in both the second and third phases of data collection (corresponding to sessions 2 and 3) than in the first, yet, sessions 2 and 3 showed more fluctuations of enjoyment in language learning. The continuous drop in Mary's enjoyment in sessions 2 and 3 due to her losing control of the passage content and meaning can be accounted by the control-value theory (Pekrun, 2006; Shao et al., 2020). In light of this theory, as Mary lost her control over both content and meaning, her levels of enjoyment reduced. Among the three phases of data collection, the third session (near the end of the course) showed the highest ups and downs in enjoyment. As the interview accounts revealed, these fluctuations were induced by different factors, mostly internal to the classroom setting and some (especially during the last phase of data collection) influenced by out-of-class conditions. This finding is consistent with the admittedly varying nature of FLE emerging influenced by different internal and external contextual factors in the ever-changing complex world of language learning pinpointed by Dewaele and Dewaele (2017, 2020) and Elahi Shirvan et al. (2020, 2021). In addition, analysis of the enjoymeter data along with the interviews revealed instances of both private and social FLE, the latter found more prevalent. Different moments of Mary's high enjoyment experience instantiated her private FLE, including her positive attitude toward language learning within the class events, especially at the outset of the course and also at the beginning of all sessions. She always began the sessions with a slightly below the average level of enjoyment. It means her enjoyment was never null at the opening of the sessions. Not all her classmates were so as, admittedly, only dragged themselves to the class and showed a lacking interest from the very beginning. However, she always brought with her a certain degree of internally initiated enjoyment she found in languagelearning experiences to class, even the last session before which she had a personal unfavorable condition at home. The other instances of Mary's FLE system reflecting the private aspect of FLE were her experiencing enjoyment when she felt capable of self-assessment, accomplishment, and a sense of development throughout the course.

The recurrent patterns in her accounts showed that she enjoyed self-teacher moments for self-assessment purposes and gaining confidence in her abilities. This is consistent with the description of the private dimension of FLE focus on personal progress and self-development (Dewaele and MacIntyre, 2014, 2016; Pavelescu and Petric, 2018).

The more social dimension of FLE (Dewaele and MacIntyre, 2016) is at the core of Mary's dynamic system of enjoyment, including her commentaries and points of view shared with interlocutors (peers and teacher), laughter in class, and pleasing relationships within and outside the classroom. Her highest moments of enjoyment marked by a sudden increase evident in the trajectories of her FLE growth happened when there was a shift to cooperative tasks in class (either in the form of student–student or teacher–student) interactions especially those meant to engage students maximally in the topic of the lesson. As Mary confessed, her teacher played a key role in effectively monitoring these interactions to maximize enjoyment.

Similarly, Daschman et al. (2014) emphasized the need for teachers to establish constructive relationships with students in class and keep them enthusiastically engaged in class activities to resist boredom. When Mary was deprived of these cooperative chances of self-expression and was given an unchallenging individual task instead (instantiated more in the last 20–30 min of the three sessions' time), she experienced a lowering trend of FLE (as manifested in the trajectory of changes). This finding can be justified by the under-stimulation theory of emotions proposed by Larson and Richards (1991), according to which low enjoyment in young adult learners can be due to any learning condition that is recurrent, habitual, and not adequately challenging. It can also significantly and negatively affect social forces too, as explored by a number of researchers, including Weibright et al. (2015).

The use of enjoymeters data in this study indicated that Mary's enjoyment in group discussions emerged under the influence of her tight social bonds with her peers. This finding might be accounted by positive peer emotion contagion (Shao and Parkinson, 2021). This contagion is supported by the integration of control-value theory and social appraisal perspective based on which the control over and value of classroom activities can mediate the influences of peers on learners' emotions (Pekrun, 2006). Thus, it seems that in the group discussions, Mary's enjoyment was transferred from her peers' feeling of enjoyment as she was observing their high control over the discussions and the high values they perceived for them. Moreover, Mary experienced instances of decreased enjoyment, as the enjoymeter data and interviews revealed, when she felt she was losing control over a given task and also left to her own devices deprived of any peer or teacher support (e.g., in the silent reading task in session 2). This lowering enjoyment induced by the loss of control over the task can be explained in the light of the control-value theory by Pekrun (2006). According to this theory, the decreased enjoyment a student feels while learning is influenced by the extent of control he thinks he has over the task as well as the value he attributes to that particular task and its content. As Mary's accounts showed, she attributed less value to the silent reading task and admitted that she preferred the teacher let them do it as a whole class activity by reading out the passage line-by-line to both check the pronunciations and the meaning of the content part by part. Though silent reading can have certain benefits for language learners, it is evident that Mary was not aware or convinced of the value of the task. Probably, if the teacher had taken time assuring her (or other students) of the value of the task, the decreasing trend of enjoyment could have been prevented. The see-saw relation between enjoyment and boredom has been already raised by other researchers. Pekrun and Linnenbrink-Garcia (2012) discussed the emotions of enjoyment and boredom in tandem in the learning process, one (enjoyment) playing an activating role while the other (boredom) playing a deactivating role regarding learning motivation. Similarly, Heckel and Ringeisen (2017) found a statistically significant negative correlation between student boredom and enjoyment in e-learning environments.

As the present findings showed, not only is it worth investigating enjoyment along with boredom in language learning, but several other personality-related or emotional constructs are worth studying in relation to FLE, including anxiety and motivation. For example, in the third session of data collection, the anxiety-provoking task significantly contributed to the decreasing trend of Mary's enjoyment. She found the task face-threatening and immediately grew concerned about it. The task which could be expected to contribute to her private FLE practically backfired and reduced enjoyment. The self-teacher interaction in this task actually neither contributed to Mary's private nor the social aspects of FLE. This is indicative of the need for a holistic approach to investigate negative and positive emotions involved in language learning inclusively, as also emphasized by Oxford (2016). As for motivation, the data analysis revealed many instances of the mutual effect of motivation and enjoyment in language learning. Part of Mary's motivation was internally oriented, especially when she compared herself with classmates who dragged themselves to class. Some other parts of her motivation were affected by the nature of tasks, as in the final Q&A task of session 1 which was demotivating and, consequently, deductive of her enjoyment. The mutual effect of motivation and enjoyment is suggested to be further explored in longitudinal studies to check for their co-development during the language learning experience.

### Pedagogical Implications of Research Findings

From a pedagogical perspective, the findings of the study, based on the application of enjoymeters, draw the attention of language teachers to the dynamic nature of FLE as it is susceptible to several momentary factors in the environment of the classroom. This means that language teachers should be sensitive to, and monitor, micro-changes in their learners' enjoyment and the factors influencing these changes, so that they can provide them with enjoyable moments of language learning in which they can perceive a high control over, and positive value of, their classroom tasks and activities.

On the other hand, given the feedback sensitive nature of dynamic systems, language teachers can nurture moments of high enjoyment for their learners through emotional scaffolding, building rapport, and group work in which they can feel a sense of belonging, enthusiasm, and connectedness (Dewaele and Li, 2020). Furthermore, enhancing playfulness among learners can enable language learners to shift from feeling negative emotions to positive ones, such as enjoyment (Barabadi et al., 2022). One type of playfulness is other-directed playfulness which can pave the way for positive peer enjoyment contagion, as a result, learners might experience more enjoyment in the classroom (Shao and Parkinson, 2021).

Moreover, based on the findings of this study, enjoymeter can be used as a tool for classroom research by language teachers, so that they can hold an ongoing assessment of their learners' enjoyment in the class. Since positive emotions can broaden cognitive capacities (Fredrickson, 2001), the ongoing assessment of language learners' enjoyment can provide teachers with appropriate feedback for effective assessment of learning.

### Limitations and Future Directions

One limitation of this study is its investigation of FLE as the outcome. Exploring FLE as both a predictor and an outcome, together, can give us a more comprehensive picture of how learner's enjoyment is affected by other factors in e-learning and how it affects other learner-related constructs or emotions experienced in this context. For instance, enjoymeters can be applied simultaneously with boredometers and anxometers in a foreign language class to see to what extent the micro-changes in enjoyment, boredom, and anxiety can co-occur. Also, the data analysis of this study was limited to thematic analysis. Other analytic approaches, such as process tracing and qualitative comparative analysis, can be used for the analysis of enjoymeter data.

In addition, enjoymeters can be used with motometers to explore the bidirectional dynamics of enjoyment and motivation within each session of a language course. Besides, as I followed the procedures of a case study, I only gained access to the information obtained from one participant. Thus, enjoymeters can be used for the exploration of the FLE dynamics as experienced by more than one learner in a foreign language class. Delving into teachers' and peers' effects on language learners' enjoyment in learning a foreign language *via* an enjoymeter complemented by journal or diary accounts can be also considered for a more inclusive social network analysis (refer to Hiver and Al-Hoorie, 2019). Moreover, exploring the dynamic quality of enjoyment in EFL learning can be done in teacher–learner dyads in which the dynamics of enjoyment in both teachers and learners and their influence on each other can be compared at the same time.

## Conclusion

This study traced the dynamic nature of language learning enjoyment in multiple phases of an EFL course in China. The findings not only contribute to the positive psychology line of research in SLA in accordance with the broaden-and-build theory (Fredrickson, 2001), but also have a methodological value as they introduce the application of an innovative measurement instrument to trace the dynamic growth of the emotional construct throughout the process of language learning. The use of the enjoymeter made it possible to follow and map the time to time and session by session growth of FLE. It showed that Mary (the participant in this study) experienced further moments of high enjoyment in the beginning of the language course than in the middle and the end of the course. On the other hand, her enjoyment in each of these three sessions changes under the influence of several factors which underpinned the emergence of both her private ELF and social ELF. Moreover, the application of enjoymeters in this study revealed that FLE behaves in a dynamic system in the ecology of classroom learning. It emerges in a non-linear path due to its feedback-sensitive nature. Teachers and peers are the two factors that influence the dynamics of social FLE. More specifically, the enjoyment experienced by peers as a result of their control over, and value of, class activities is contagious and can play a pivotal role in the emergence of FLE.

## Data Availability Statement

The raw data supporting the conclusions of this article will be made available by the authors, without undue reservation.

## Ethics Statement

Ethical review and approval was not required for the study on human participants in accordance with the local legislation and institutional requirements. The patients/participants provided their written informed consent to participate in this study.

## Author Contributions

RW developed the research idea and writing of the whole manuscript.

## Conflict of Interest

The author declares that the research was conducted in the absence of any commercial or financial relationships that could be construed as a potential conflict of interest.

## Publisher's Note

All claims expressed in this article are solely those of the authors and do not necessarily represent those of their affiliated organizations, or those of the publisher, the editors and the reviewers. Any product that may be evaluated in this article, or claim that may be made by its manufacturer, is not guaranteed or endorsed by the publisher.

## Appendix: Enjoymeter

**Table A1:** Rate the intensity of enjoyment you experience on the following 0–10 scales: How joy did you feel in each part of the class time?

10	10	10	10	10	10	10	10
9	9	9	9	9	9	9	9
8	8	8	8	8	8	8	8
7	7	7	7	7	7	7	7
6	6	6	6	6	6	6	6
5	5	5	5	5	5	5	5
4	4	4	4	4	4	4	4
3	3	3	3	3	3	3	3
2	2	2	2	2	2	2	2
1	1	1	1	1	1	1	1
0	0	0	0	0	0	0	0
							
**0-10**	**10-20**	**20-30**	**30-40**	**40-50**	**50-60**	**70-80**	**80-90**
